# Mycotoxins and Antifungal Drug Interactions: Implications in the Treatment of Illnesses Due to Indoor Chronic Toxigenic Mold Exposures

**DOI:** 10.1100/tsw.2004.22

**Published:** 2004-03-12

**Authors:** Ebere C. Anyanwu, Andrew W. Campbell, John E. Ehiri

**Affiliations:** ^1^ Cahers Neurosciences Research, Inc., 8787 Shenandoah Park Drive, Suite 122, Conroe, TX 77385, USA; ^2^ Medical Center for Immune, Environmental, and Toxic Disorders, Spring, TX, USA; ^3^ Department of Maternal and Child Health, School of Public Health, University of Alabama at Birmingham (UAB), Ryals Public Health Building, 1665 University Boulevard, Birmingham, AL 35294-0022, USA

**Keywords:** water-damaged buildings, toxigenic molds, mycotoxins, antifungal drugs

## Abstract

Chronic exposure to toxigenic molds in water-damaged buildings is an indoor environmental health problem to which escalating health and property insurance costs are raising a statewide concern in recent times. This paper reviews the structural and functional properties of mycotoxins produced by toxigenic molds and their interactive health implications with antifungal drugs. Fundamental bases of pathophysiological, neurodevelopmental, and cellular mechanisms of mycotoxic effects are evaluated. It is most likely that the interactions of mycotoxins with antifungal drugs may, at least in part, contribute to the observable persistent illnesses, antifungal drug resistance, and allergic reactions in patients exposed to chronic toxigenic molds. Safe dose level of mycotoxin in humans is not clear. Hence, the safety regulations in place at the moment remain inconclusive, precautionary, and arbitrary. Since some of the antifungal drugs are derived from molds, and since they have structural and functional groups similar to those of mycotoxins, the knowledge of their interactions are important in enhancing preventive measures.

## INTRODUCTION

Water-damaged buildings are known to be susceptible to contamination by toxigenic molds, which can expose the occupants to mycotoxins released into the indoor environment by the toxigenic molds[1]. Consequently, the issue of water-damaged buildings has attracted much attention in recent times because of the escalating health and property insurance costs nationwide. In the last 20 years or so, an increasing number of associated illnesses with potentially fatal outcomes in children and older adults has been reported[1]. The most common mycotoxin-producing molds that have been implicated in these illnesses include *Fusarium, Trichoderma, Stachybotrys, Cladosporium, Penicillium, Aspergillus*, and *Alternaria*[2,3]. They have the potential to produce extremely potent secondary metabolites, some of which are beneficial (antibiotics) and some of which are toxic (mycotoxins) to humans and animals. They are natural contaminants of cereals and other edible commodities throughout the world. The main medium of human toxigenic mold infection is through inhalation of the spores, and the most common route is ingestion, but it may also involve dermal, respiratory, and parenteral routes, the last being associated with drug abuse[4,5,6,7]. Although some efforts have been made by different government environmental health agencies to regulate the presence of mycotoxins in foods, there is still a huge problem concerning the specific effects of different mycotoxins released by several toxigenic molds that are identified with one another (ecologically compatible), each affecting the structural, biochemical, and functional activity of the other[8]. Understanding the human effects of mycotoxins and antifungal drug interactions is, therefore, the first step forward and of great value in preventing and treating various illnesses associated with chronic toxigenic mold exposures[9,10]. Furthermore, because there are no specific clinical tools for assessing the dose effect of mycotoxins, it is the aim of this paper to review the structure and functions of mycotoxins, their relationships, and the implications in the treatment of fungal infections. It is hoped that these would form the basis for further research work.

## Mycotoxins

Mycotoxins are toxic chemicals released by indoor toxigenic molds. Under favorable physiological conditions, they undergo metabolic processes to produce yet other toxic chemical compounds known as volatile organic compounds (VOCs), which are responsible for the moldy odors often perceived in contaminated indoor environments. There are already several mycotoxins produced by toxigenic molds (Table 1), some of which have been studied extensively. Mycotoxins are lipid soluble and are absorbed readily by the intestinal lining, airways, and skin. Their toxic effects are thought to be associated with exposure to toxins on the surface of the mold spores and not with the growth of the mold in the body. Some toxigenic molds are capable of producing one or more mycotoxins that are limited to particular genera (specific), while others can produce the same mycotoxins that are not specific to any genera (common).

**TABLE 1**
**A List of Some of the Known Mycotoxins**

| | | |
|---|---|---|
| Aflatoxins | Fusaric Acid | Penicillic Acid |
| Alternariol | Fusariocin | Penitrem |
| Citreoviridin | Fusarins | Phomopsin |
| Citrinin | Islanditoxin | Roridins |
| Cyclopiazonic Acid | Luteoskyrin | Rubratoxin |
| Deoxynivalenol | Moniliformin | Rubratoxin |
| Diacetoxyscirpenol | Monoacetoxyscirpenol | Slaframine |
| Dicoumarol | Neosolaniol | Sporidesmin |
| Ergotamine | Ochratoxins | Stachbotryotoxins |
| Ergo Toxins | Oosporein | Sterigmatocystin |
| Fumitremorgen | Paspalitrems | T-2 Toxin, Tremorgens |
| Fumonisins | Patulin | Zearalenone |

The genera-specific and genera-common mycotoxins are presented in Table 2. In the genera-common mycotoxins, *Aspergillus* species produce aflatoxins and Sterigmatocystin. Aflatoxins are perhaps the best-known and most researched mycotoxins in the world. *Aspergillus* and *Penicillium* produce four genera-common mycotoxins, including citrinin (nephrotoxic), ochratoxin (carcinogenic), patulin, and gliotoxin (cytotoxic and immunosuppressive effects). *Penicillium* alone produces *Cladosporium cladioporioides* and emodin. Emodin has antibiotic properties as well as mycotoxic effects. *Penicillum* and *Memnoniella echinata* commonly produce griseofulvin. Grieseofulvin exhibits tumorgenic, carcinogenic, tetratogenic, and hepatotoxic reactions. Cyclopiazonic acid is produced by *Penicillium* and *Aspergillus*[11]. *Tolypocladium inflatum* specifically produce cyclosporin A. Cyclosporin A is reported to be immunosuppressive and a potential carcinogen, and used in medicine after organ transplants for the prevention of organ rejection[12].

**TABLE 2**
**A List of Toxigenic Molds and Specific and Common Mycotoxins**

| Type of Mold | Mycotoxin | Extent of Production |
|---|---|---|
| *Aspergillus flavus* and other *Aspergillus* species | Aflatoxins<br>Sterigmatocystin | Specific |
| *Aspergillus* + *Penicillium* | Citrinin<br>Ochratoxin<br>Patulin<br>Gliotoxin | Common |
| *Penicillium* + *Cladiosporium* | Emodin | Common |
| *Penicillium* + *Memnoniella* | Griseofulvin | Common |
| *Tolypocladium inflatum* | Cyclosporin | Specific |
| *Fusarium* species | Deoxynivalenol (or vomitoxin)<br>Fumonisin B1<br>T-2 toxin | Specific |
| *Stachybotrys chartarum* | Satratoxin H | Specific |
| *Myrothecium* + *Stachybotrys* | Roridin A + 3<br>Isomers | Common |
| *Myrothecium* | Verrucarin A | Specific |

*Fusarium* produces high concentrations of trichothecenes, ochratoxin A, zearalenone, deoxynivalenol (also known as DON or vomitoxin), fumonisin B1, and T-2 toxin[13,14,15]. Deoxynivalenol has been found to cause reduced weight gain, suppressed feeding, and at higher concentration levels, there can be an emetic response in animals and humans[14,15]. T-2 toxin (a trichothecene) induces hemorrhagic, immunosuppressive reactions, nausea and vomiting, DNA damage, and cell death[15]. *Stachybotrys* and *Myrothecium* produce roridin A. Roridin A is a trichothecene mycotoxin that has a cosmopolitan distribution. *Myrothecium* specifically produces verrucarin A mycotoxin. Verrucarin A is a macrocyclic trichothecene triester of the sesquiterpene alcohol verrucarol, and is synonymous with muconomycin A. They are both closely related to roridin mycotoxin. Specifically, some strains of *S. chatarum* produce satratoxin H that is implicated in very high cytotoxicity and several environmental allergic reactions.

## The Structural Properties of Mycotoxins

Mycotoxins are complex organic chemical compounds with molecular weights generally greater than 200 mass units produced when the mycelium is subjected to nutrient limitations during growth[16]. Most of these mycotoxins are structurally related to one another[17]. A single mold is capable of producing several mycotoxins that structurally have the propensity to cause harm to humans. With the aid of a combination of X-ray crystallographic analysis, NMR spectroscopy, and chemical transformations, it has been shown that mycotoxins undergo the process of isomerism. *M. verrucaria* for example, produces all four roridin E isomers, diastereomeric at the C-6' and C-13' centers, the exceptions being those congeners having a C-4' ketone group in the macrolide ring[18]. A representative structure of mycotoxins is shown in Fig. 1. Generally, they are a complex macrocyclic group some of which consist of a series of sesquiterpene polyalcohols, esters, and several ergosterol epoxides[19,20,21].

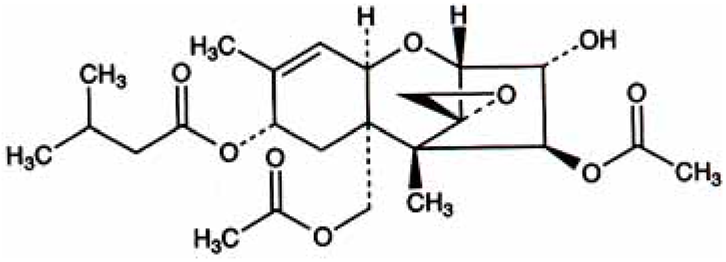

**FIGURE 1**. Representative molecular structure of trichothecene: T-2 toxin.23.

## Physiological Properties

In general, mycotoxins exert various biological activities, including insecticidal, antifungal, antibacterial, and antiviral. They are also known to exhibit phytotoxic and cytotoxic qualities, and inhibit a high degree of cytostaticity; the latter property makes them attractive antitumor candidates. Mycotoxins have also been associated with a wide variety of human and animal intoxications throughout the world[19,20,21,22,23].

## Electrophilic Properties

Although the electrophilic properties of mycotoxins have not been documented fully, there are some indications that mycotoxins in general have electrophilic properties. Such indications are found in the published works of Fliege and Metzler on the electrophilic properties of patulin[24,25,26]. In these reports, the electrophilic properties and nonenzymatic reaction of patulin with thiol-containing tripeptide glutathione and metabolic degradation product N-acetyl-L-cysteine (NAC) were demonstrated. The major products formed in the reaction of PAT with glutathione (GSH) were of the same structural type as obtained with NAC. Mycotoxin patulin was also found to induce intra- and intermolecular protein cross-links *in vitro* involving cysteine, lysine, and histidine side chains, and alpha-amino groups[25,26].

## Antibiotic Properties

Some mycotoxins (e.g., blasticidin A and citrinin) have antibiotic properties that show strong inhibitory activity toward other mycotoxins production by significantly reducing the expression of genes encoding their biosynthetic enzymes and regulatory genes[27,28]. In the studies of the antibiotic and toxigenic properties of 106 mold strains[29,30], which were the potential producers of macrocyclic trichothecenes, it was shown that 40% had high antibiotic activity. Others, such as *Laetisaria fuciformis* molds, produce diffusible compounds that are inhibitory to a range of Gram-positive and -negative bacteria but not to the mould species tested[29,31,32,33].

## Dose Effects of Mycotoxins — How Safe Are They?

There is no evidence in the literature to demonstrate the dose levels of mycotoxin(s) that could be safe to humans. Whatever regulation available now is purely precautionary and arbitrary. The determination as to whether or not a given concentration of mycotoxin is safe will depend on a number of factors including chemical class and chemical structure of the mycotoxins. The length of time of exposure to the mycotoxin(s) may vary from a few days to several years. It is assumed from animal experimental derivations that the risks of mycotoxigenesis increase as exposure time increases. In humans, however, no observable effect levels (NOEL) for mycotoxins exists. The adult LD50 (lethal dose to 50% of exposed individuals) of trichothecenes is estimated to be only 35 mg[34]. As the research interest in mycotoxicity evolves, more and more mycotoxins are identified and the unsolved problems of dose effect continuously complicate our systematic understanding of the pathogenesis of mycotoxins. The complication stems, at least in part, from the fact that mycotoxins have functional groups that exert different health effects on both humans and animals. Their metabolites are reported more frequently as potent carcinogenic substance known, and if one chemical bond is changed in the structure of the molecule, its toxicity can be reduced dramatically.

## Is Mycotoxic Effect Age and Dose Dependent?

Another important question is whether there is any tolerable dose and whether mycotoxic effects are age and dose dependent. In this case, there are no specific answers because of the multiplicity of mycotoxins released by a single mold and the fact that many molds release similar mycotoxins. From the available literature that pertain to “fungal drug” use, it appears as though age and dose inferences are based mostly on animal models of mycotoxicity, and little of humans. Thuvander et al.[35] estimated the intake of some mycotoxins from food in Sweden and found that mycotoxin levels in the food samples were generally below the European/national maximum limits. However, the intake of ochratoxin A, patulin, and trichothecenes was found to be below the temporary daily intake values (tTDI) proposed for these toxins by international expert groups. Although the intake of trichothecenes (expressed as T-2 toxin equivalents) in children with a high consumption of cereals was close to the tTDI for T-2 toxin, such a value would be seriously unreliable[35,36,37].

## Pathophysiological Implications

The symptoms of mycotoxin poisoning largely are dependent on the type of mycotoxin, the dose, route of exposure, and the sensitivity of the person exposed. Here are just a few of the many short-term symptoms that have been documented in the scientific and medical literature. The effects of acute exposures to toxigenic molds could occur within hours or days of exposure and may lead to headaches, coughing, skin rashes, fever, nausea, sore throat, nosebleeds, congestion, fatigue, depression, and diarrhea. However, chronic exposures may lead to kidney and respiratory disorders, liver cancer, lung disease, lung cancer (glotoxin, aflatoxin), brain disease (citroviridin, satratoxin), immune suppression, skin disease, respiratory disorders, birth defects, and organ damage (trichothecenes, zearalenone)[38,39,40].

In general, the severity of the pathophysiological effects of mycotoxins probably depends, at least in part, on the concentration of toxic molds in the environment, the type of the metabolites produced, the dose effect of mycotoxins in the exposed individuals, and the individual constitutional variability. It has been observed in our Center that the dominance of a particular species of toxigenic molds such as *Penicillium* may influence the pathophysiological effects of other fungal mycotoxins in the patients’ serum. Hence, whenever there is one or no *Penicillium* species present, for example, the concentrations of all other toxigenic molds are affected. In other words, it could be that the mycotoxins from one dominant toxigenic species could suppress the activity of the mycotoxins from other molds by masking the reactive regions of such mycotoxins[34,41]. Besides health effects such as headaches, fatigue, and respiratory diseases, mycotoxins have been shown to be carcinogenic in laboratory animals[8,42]. The mechanisms for cancer induction by mycotoxins include the possible role of oxidative damage during initiation and the disruption of lipid metabolism, integrity of cellular membranes, and altered growth-regulatory responses as important events during promotion[42]. Lipid peroxidation is one of the main manifestations of oxidative damage and has been found to play an important role in the toxicity and carcinogenesis of many carcinogens such as mycotoxins[43].

## Problems of "Undesired" Toxicological Effects

One of the problems in elucidating the mechanism of mycotoxicity is being able to determine the minimal tolerable blood level in humans. The mycotoxin dose levels, the ligand-receptor, and their complexes are not yet known. However, it is assumed that if the ligand is endogenous, the effect will have a physiologically undesired toxicological effect. In addition, if the reaction is not endogenous, the effects are expected to be pharmacological (Fig. 2). For a mycotoxicity to have toxicologically undesired effects, three major manifestations must be observed in both adult and children, either at short- or long-term endpoints.

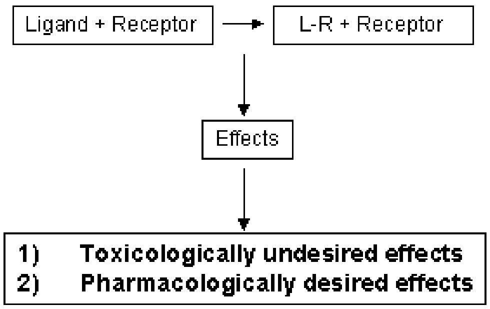

**FIGURE 2.** Proposed basic mechanism of mycotoxic effects.

Behavioral functions (adults) such as impaired memory and learning ability must be affected. The problem here is that the concentration to which behavioral changes are effected is not yet determined. Furthermore, changes in individual constitution and genetic make up may affect the levels of behavioral changes.

Peripheral nerve conduction functions in adults must be affected, i.e., there should be a decreased nerve conduction velocity. The question here is: At what concentration is nerve conduction velocity affected? Is the effect transient? What neurological signs and symptoms are manifested in children? If these signs and symptoms are at high levels of concentration, can the possibility of encephalopathy be ruled out?

## Neurodevelopmental and Pharmacologically Desired Effects

Neurological effects of mycotoxins are those effects on the structure or functioning of the central nervous system (CNS) or peripheral nervous system that result from exposures to toxigenic molds. They can cause morphological changes that lead to generalized damage to nerve cells (neuropathy), injury to axons (axonopathy), destruction of the myelin sheath (myelinopathy), or neurobehavioral dysfunction[44,45,46]. If mycotoxins affect development, it then implies that the anatomical and functional "hard wiring" and differentiation in the CNS may be affected. There could be an overexpression of the proteolipid protein (PLP) gene, endosomal/lysosomal accumulation of cholesterol, and this accumulation is shown to perturb the process of myelination and impair the viability of oligodendrocytes[47]. Impairment of oligodendrocytes and myelin could lead to the molecular cascade leading to white matter damage[48]. If that happens, the adverse effects may occur during synaptogenesis. That means that cell:cell adhesion may be affected too[49]. On the other hand, if a mycotoxin has a pharmacological effect, the cellular homeostasis and the ionic mechanism of neurotransmission may be affected. These effects may either lead to the inhibition or facilitation of transmitter release and modulation of ionic conductance. In children for example, mycotoxins affect memory and attention, and in adults mycotoxins produce peripheral neurotoxicity, while in the CNS they affect mood and effects[50]. However, as far as developmental and pharmacological effects are concerned, further questions are asked:

- Are these changes related to each other and how?
- Which molecular actions of mycotoxins are related to one or more of these effects?
- Are neurotransmitter release and transmitter-gated ion channels only at high concentration in the processes of mycotoxicity?
- How do mycotoxins enter neurons and affect intracellular sites such as second messenger and ion-storage system?
- What are the mycotoxin binding proteins in the brain that may help to determine their toxicity?
- What cellular event is most sensitive to mycotoxin activation of protein kinase C, *in vitro* or *in vivo*, at what concentration?
- Can the mitochondrial release of Ca2+-sensitive nM range of mycotoxin be measured?
- Is DA-sensitive adenylate cyclase sensitive to µM range of mycotoxin; is Na/ATPase also sensitive to mycotoxins, probably to very low concentration?
- Are there any phosphorylation reactions associated with learning and do these mechanisms connect with behavior/learning?

Until these questions are answered fully through compelling evidence-based research outcomes, the understanding of the cause-effects of mycotoxins and their interactions with antifungal drugs will remain a serious health problem.

## Treatment of Illnesses Due to Toxigenic Mold Exposures

Consequently, treatment of diseases due to toxigenic mold exposures is a complex endeavor that requires proper knowledge and extensive experience of the nature and behavior of the molds. Most of the antifungal drugs are derived from the mycotoxins, for example, the antifungal polyenes (amphotericin B, fungizone), griseofulvin, flucytosine, azoles (imidazoles and triazoles), allylamines, and others. Most of them have similar chemical and physical properties to mycotoxins[51]. Itraconazole, the azole derivative that is active against *Aspergillus*, may be administered only in mild cases of *Aspergillus* infections in immunocompromised patients. Although, intravenous amphotericin B (AMB) and oral itraconazole remain the standard of care for invasive aspergillosis, their effectiveness against other mycotoxins is not fully established. The overall response rate was 39%, in other words, there are variable results in treating patients with amphotericin B, itraconazole, or both[51]. Therefore, in treating fungal infections, the structural and preferential effectiveness must be considered.

### Structural Consideration

A number of antifungal drugs have the ability to degrade and inactivate certain mycotoxins. They do so by mechanistically “masking” the functional groups or degrading mycotoxins to their nonactive state through the processes of ammoniation and sulfitization[52,53,54]. What this means, for example, is that those mycotoxins that do not contain amino and sulfite functional groups (trichothecenes) can easily respond to antifungal drugs that contain amino and sulfite groups (acid-base reaction). The mechanism for this action appears to involve hydrolysis of the lactone ring and subsequently converts the parent mycotoxin compounds to numerous products that exhibit greatly decreased toxicity[52,53,54,55].

### Preferential Effectiveness Consideration

Certain toxigenic molds dominate and exert inhibitory effects on the activities of other molds. Since most antifungal drugs are derived from mycotoxins, it then implies that their effectiveness will probably depend on the predominant mold mycotoxins present in the patients’ serum. The antifungal drug would interact preferentially with the mycotoxins for which it has inhibitory affinity. For example, if a patient is presenting with infections due to mycotoxins with azole structure, and is treated with the antifungal azole drugs, the clinical response may not be a 100% effective. Although the patient’s condition may apparently improve slightly, the azole mycotoxin will relapse with time, as we have observed in our Center.

Another example is in the case of fluconazole and clotrimazole that are the most commonly prescribed treatments for oral candidiasis. One would find that the two drugs are functionally similar and the difference between their effectiveness monotherapeutically and polytherapeutically is not much. Hence, the clinical response from two groups of patients[56] treated with both drugs was statistically equivalent in both groups (98% of fluconazole recipients and 94% of clotrimazole recipients). However, the efficacy of elimination of *Candidiasis albicans* with fluconazole was 65%, while clotrimazole was 48%, and when two antifungal drugs with different functional groups are compared, significant differences were possible[57,58,59]. In addition, separate treatment of patients with itraconazole 200 mg daily and ketoconazole 200 mg daily showed no significant difference in time to clearing infection between the two treatments[60,61]. The differences in these cases may be due to the types of unrelated mycotoxins present in the patient’s serum, and not necessarily on the superiority or efficacy of the individual drugs[60,61,62].

## CONCLUSIONS

Mycotoxins, antifungal drug interactions, and the implications in the treatment of illnesses due to indoor chronic toxigenic mold exposures have been reviewed. It is very important that the structural and functional relationships of the mycotoxins with one another and with antifungal drugs be understood fully so that a proper treatment strategy could be carried out with a higher level of confidence. It is suggested appropriately that if a patient is sensitive to those mycotoxins that can be produced by *Penicillium* species, for example, then it would not be advisable to prescribe the patients with penicillin medication of any type, otherwise the patient would be at risk of adverse reaction to the penicillin. It is important also that the identities, tolerable doses, and long- and short-term adverse effects of the mycotoxins be determined to avert or minimize the scenario of adverse antifungal drug reactions.

## BIOSKETCHES

**Ebere C. Anyanwu, PhD,** is a Clinical Neurophysiologist and Director of Cahers Neurosciences Research, Inc., Conroe, TX. He is also an Adjunct Professor of Anatomy and Physiology at the North Harris and Montgomery Colleges in Houston and The Woodlands, respectively. E-mail: ebereanyanwu@msn.com

**Andrew W Campbell**, **MD**, is an Expert Medical Scientist in Immunotoxicology and the Medical Director and Chief Executive of the Medical Center for Immune, Environmental and Toxic Disorders, Houston, TX. E-mail: md@immunotoxicology.com

**John Ehiri, PhD, MPH,** is an Assistant Professor in the Department of Maternal and Child Health, School of Public Health, University of Alabama at Birmingham (UAB), Birmingham, AL. E-mail: jehiri@uab.edu.
